# Prognostic value of lymph node density on cancer staging system for gastric cancer without distal metastasis: a population-based analysis of SEER database

**DOI:** 10.1186/s12957-022-02795-9

**Published:** 2022-09-29

**Authors:** Yuhua Liu, Hao Cui, Xinxin Xu, Wenquan Liang

**Affiliations:** 1 Chinese PLA General Hospital & Medical School of Chinese PLA, Beijing, 100853 China; 2 Institution of Hospital Management, Department of Medical Innovation and Research, Chinese PLA General Hospital, Beijing, 100853 China; 3 Department of General Surgery & Institute of General Surgery, the First Medical Center of Chinese PLA General Hospital, Beijing, 100853 China

**Keywords:** Lymph node density, Gastric cancer, SEER, Tumor staging, Overall survival, Cancer-specific survival

## Abstract

**Background:**

Accurate tumor staging is the cornerstone of tumor treatment. Current tumor staging system for gastric cancer (GC) is based on regional positive lymph nodes while ignoring the total number of examined lymph nodes. We aim to assess the prognostic value of lymph node density (LND), the ratio of positive nodes to the total number examined nodes, in GC without distal metastasis.

**Methods:**

Clinical information of patients with histologically confirmed GC and without distal metastasis was identified from the Surveillance, Epidemiology, and End Results (SEER) database between 2010 and 2015. The X-Tile software was used to identify the ideal prognosis-related cutoff point for LND. The prognostic value of LND on cancer-specific survival (CSS) and overall survival (OS) was assessed in Cox regression models. Subgroup analysis stratified by LND was performed on current lymph node staging system to further explore the interaction between LND and current lymph node staging system.

**Results:**

A total of 4281 participants were identified from the SEER database for the final analysis. The optimal prognosis-related cutoff values of LND were calculated as 0.1 and 0.4, and LND was divided into three levels: LND1 (< 0.1), LND2 (> = 0.1, < 0.4), and LND3 (> = 0.4). LND3 was associated with worse CSS and OS in GC patients. Compared to patients with LND1, those with LND2 and LND3 had 2.43 (HR = 2.43, 95% CI 2.09–2.84, *P* < 0.001) and 4.69 (HR = 4.69, 95% CI 4.02–5.48, *P* < 0.001) folds increase in mortality in CSS, respectively. Similar results were found in the evaluation of OS in GC patients. Subgroup analysis stratified by LND also found that patients in the same current lymph node stage still had different prognosis due to the different LND levels after adjustment for other prognosis-related covariates (all *P* values < 0.001).

**Conclusion:**

LND is an independent prognostic factor for GC without distal metastasis. In the current lymph node staging system, LND has potential value in further accurately classifying GC patients without distal metastasis.

**Supplementary Information:**

The online version contains supplementary material available at 10.1186/s12957-022-02795-9.

## Background

Gastric cancer (GC) is the fourth most commonly diagnosed cancer on the upper digestive system and remains the second most common cause of cancer-related deaths worldwide [1]. Cancer staging is the cornerstone of successful tumor therapy after surgery. Today, the American Joint Committee on Cancer (AJCC) tumor-node-metastasis (TNM) classification is the most widely used staging system for GC [2–4]. Lymph node staging system, also defined as N category, is an essential factor for predicting prognosis and selecting the appropriate treatment [5]. In the AJCC staging system, only the number of positive regional nodes is taken into account and the number of the total examined regional nodes does not get enough attention [6, 7]. However, GC patients with same number of regional positive nodes accompanying with more total regional nodes examined tend to have a higher level of radical gastrectomy and lower risk with the residual tumor [8, 9]. New attempt and exploring an evaluating criterion of the lymph node staging system is needed.

Several studies have demonstrated that GC patients with more examined regional nodes were more likely to have better survival because they tended to have a higher level of radical gastrectomy [10–12]. With the development of surgical techniques, obtaining and examining an increasing number of total regional nodes is no longer difficult for an experienced surgeon. Lymph node density (LND), also called lymph node ratio (LNR), is calculated by the ratio of the number of positive nodes to the total examined nodes [13, 14]. This parameter has been proven as a potential prognostic factor in several cancers, including GC [15, 16]. From the 7th edition (the year 2010) of the AJCC staging system, resection of at least 16 regional lymph nodes was introduced into the guideline of GC surgery [17]. The number of lymph node dissection before 2010 was small, usually less than 15, and previous studies of LND in GC were limited because they enrolled the patients both before and after 2010 [18]. The prognostic value of LND needs to be reassessed by only using data after 2010, which is same as the current lymph node dissection standard.

In the present study, we determined the impact of LND on cancer-specific survival (CSS) and overall survival (OS) in GC patients based on data after 2010 in the Surveillance, Epidemiology, and End Results (SEER) database. We hypothesize that LND is an independent prognostic factor in GC. The interaction between LND and current lymph node staging system was detected to provide evidence for the development of a novel accurate lymph node staging strategy.

## Methods

### Patients and data

**Fig. 1:**
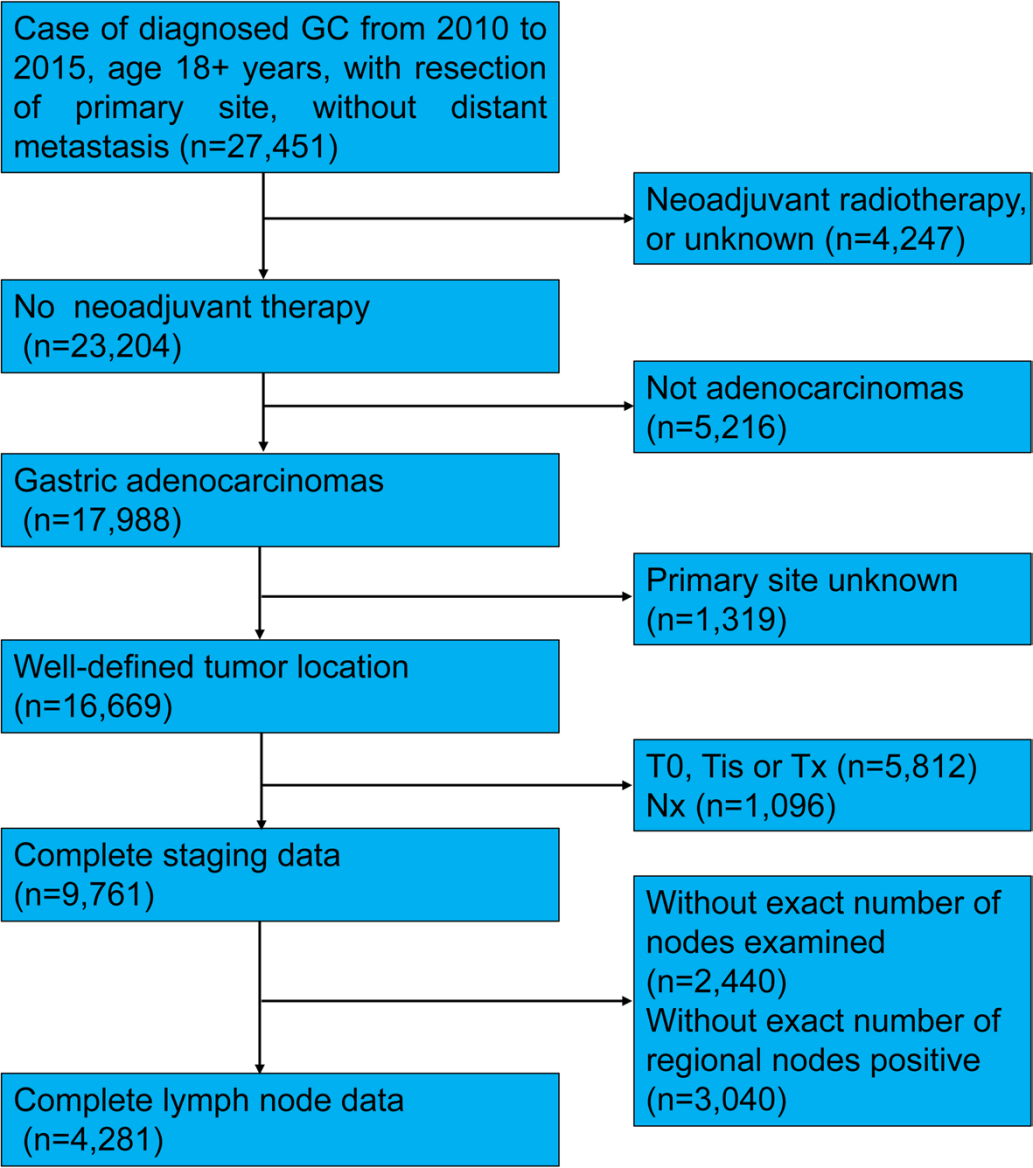
Flow chart illustrating the screening process in this study

This study had a retrospective design and included data from patients with histologically confirmed GC from the SEER database. Data were recorded according to the third edition of the International Classification of Diseases for Oncology. Enrollment occurred between 2010 and 2015, during which period the 7th AJCC staging strategies were applied and the follow-up and treatment information was complete. Patients were included if they were older than 18 years and had undergone gastrectomy. Patients with neoadjuvant radiotherapy were removed from the analysis, and only those with a histological type of adenocarcinoma were enrolled. Patients with incomplete data regarding tumor location or staging information were also excluded from the analysis. The primary variable in this study was lymph node data and patients lacking information regarding the exact number of nodes examined or positive nodes were not included in the analysis. In the 7th edition (2010) of the AJCC staging system for gastric cancer, the cutoff levels of N category were as follows: N0 = 0 node; N1 = 1–2 nodes; N2 = 3–6 nodes; N3 = more than 6 nodes. The patient screening process is shown in Fig. 1. Finally, a total number of 4281 patients with GC were included for analysis in the present study.

The following available information on pathological and clinical appearance was collected from data-mining: age at diagnosis, sex, year of diagnosis, race, marital status at diagnosis, insurance status, primary site, 7th AJCC stage group information, grade, chemotherapy, lymph node data, cause-specific death classification, vital status recode, and survival months. Overall survival (OS) was determined with the vital status recode and survival months, while cancer-specific survival (CSS) was calculated with the cause-specific death classification and survival months. Informed consent was not obtained for the present study because of the open-source nature of the SEER database. This study was conducted in accordance with the Declaration of Helsinki.

### Statistical analyses

LND was calculated by the ratio between the number of positive nodes and the number of examined nodes. Means were calculated for continuous variables and proportions for categorical variables. To identify the ideal cutoff point of LND in predicting survival, the X-Tile software (3.6.1 version, Yale University) was used [19]. The chi-square test (categorical variables), one-way analysis of variance (normal distribution), or the Mann-Whitney *U* test (skewed distribution) was used to test for differences among different LND groups. The relationship between specific variables and survival were analyzed by the Kaplan-Meier log-rank test and Cox proportional hazards regression. Univariate analyses of individual pathological and clinical features and CSS or OS were performed. To identify any independent association between LND and survival, we constructed three models: non-adjusted model, where no covariates were adjusted; adjusted I model, where only sociodemographic data of age, sex, and race were adjusted and adjusted II model, where covariates in the adjusted I model and other elected covariates were adjusted. If the matched odds ratio changed at least 10% as the result of adding covariates, the variables were considered to be added in the adjusted II model, as described in a previous study [20]. To estimate the robustness of LND in predicting survival, sensitivity analyses were stratified according to different AJCC N stages. Tests for effect modification for those of subgroup indicators were followed by the likelihood ratio test. All the analyses were performed with the statistical software package R (http://www.R-project.org, The R Foundation) and EmpowerStats (http://www.empowerstats.com, X&Y Solutions, Inc., Boston, MA). *P* values less than 0.05 (two-sided) were considered statistically significant.

## Results

### Demographic characteristics and relationship between LND and clinical features

After screening by inclusion and exclusion criteria, a total of 4281 participants diagnosed with GC were selected from the SEER database for the final data analysis. Table 1 provides an overview of patient characteristics and clinicopathologic features. The average age at diagnosis was 69.73 years for the whole population enrolled, and almost two-thirds of the participants (63.78%) were male. The number of enrolled patients was evenly distributed throughout the enrollment period from 2010 to 2015. Other baseline demographic data and information on pathologic staging and grading are shown in Table 1. The X-Tile software was used to identify the ideal cutoff point for LND in predicting survival. As shown in Supplemental Figure S1 and S2, cutoff points of 0.1 and 0.4 yielded the maximum chi-square of log-rank tests for CSS and OS, and LND was divided into three levels: LND1, LND2, and LND3. The survival difference was significant in the three groups both for CSS and OS when divided by the best cutoff points. The relationship between different groups of LND and clinical features is shown in Table 1. Insured patients tended to have a lower level of LND than that the uninsured or unknown categories, indicating that the insured population may have easier access to physical examinations and thus receive an earlier diagnosis. Tumors located on the upper stomach tended to have a lower level of LND and tumors with overlapping lesions tended to have a higher level of LND. A high rate of LND in advanced AJCC staging and grading is clearly shown in Table 1. This result is somewhat counterintuitive and requires further investigation since patients in the middle level of LND were more likely to receive chemotherapy.

**Table 1:** Patient characteristics and correction between LND and clinicopathologic variables

Variables	Total	LND1 (< 0.1)	LND2(> = 0.1, < 0.4)	LND3 (> = 0.4)	*P* value
Patients, n	4821	3170	871	780	
Regional nodes examined	17.96 ± 12.67	17.63 ± 12.64	20.02 ± 12.65	17.01 ± 12.58	< 0.001
Regional nodes positive	2.69 ± 5.40	0.20 ± 0.57	4.27 ± 3.14	11.09 ± 8.32	<0.001
Age at diagnosis (years)	69.73 ± 12.30	69.47 ± 11.98	70.18 ± 12.65	70.24 ± 13.12	0.143
Sex					0.291
Male	3075 (63.78%)	2033 (64.13%)	536 (61.54%)	506 (64.87%)	
Female	1746 (36.22%)	1137 (35.87%)	335 (38.46%)	274 (35.13%)	
Year of diagnosis					0.225
2010	842 (17.47%)	536 (16.91%)	170 (19.52%)	136 (17.44%)	
2011	816 (16.93%)	523 (16.50%)	143 (16.42%)	150 (19.23%)	
2012	826 (17.13%)	558 (17.60%)	138 (15.84%)	130 (16.67%)	
2013	780 (16.18%)	500 (15.77%)	147 (16.88%)	133 (17.05%)	
2014	800 (16.59%)	527 (16.62%)	147 (16.88%)	126 (16.15%)	
2015	757 (15.70%)	526 (16.59%)	126 (14.47%)	105 (13.46%)	
Race					0.229
White	3121 (64.74%)	2024 (63.85%)	573 (65.79%)	524 (67.18%)	
Asian or Pacific islander	1019 (21.14%)	692 (21.83%)	180 (20.67%)	147 (18.85%)	
Black	629 (13.05%)	413 (13.03%)	113 (12.97%)	103 (13.21%)	
Other	52 (1.08%)	41 (1.29%)	5 (0.57%)	6 (0.77%)	
Marital status at diagnosis					0.068
Married	2840 (58.91%)	1900 (59.94%)	499 (57.29%)	441 (56.54%)	
Widowed	738 (15.31%)	474 (14.95%)	140 (16.07%)	124 (15.90%)	
Single	624 (12.94%)	377 (11.89%)	126 (14.47%)	121 (15.51%)	
Other	619 (12.84%)	419 (13.22%)	106 (12.17%)	94 (12.05%)	
Insurance					0.012
Insured	2950 (61.19%)	1981 (62.49%)	526 (60.39%)	443 (56.79%)	
Uninsured or unknown	1871 (38.81%)	1189 (37.51%)	345 (39.61%)	337 (43.21%)	
Primary site					< 0.001
Upper	1300 (26.97%)	931 (29.37%)	230 (26.41%)	139 (17.82%)	
Middle	1433 (29.72%)	955 (30.13%)	254 (29.16%)	224 (28.72%)	
Lower	1702 (35.30%)	1092 (34.45%)	298 (34.21%)	312 (40.00%)	
Overlapping lesion	386 (8.01%)	192 (6.06%)	89 (10.22%)	105 (13.46%)	
AJCC stage group, 7th (2010-2015)					< 0.001
Stage I	1910 (39.62%)	1877 (59.21%)	27 (3.10%)	6 (0.77%)	
Stage II	1287 (26.70%)	1010 (31.86%)	207 (23.77%)	70 (8.97%)	
Stage III	1624 (33.69%)	283 (8.93%)	637 (73.13%)	704 (90.26%)	
AJCC T, 7th (2010-2015)					<0.001
T1	1644 (34.10%)	1539 (48.55%)	80 (9.18%)	25 (3.21%)	
T2	708 (14.69%)	554 (17.48%)	98 (11.25%)	56 (7.18%)	
T3	1577 (32.71%)	829 (26.15%)	416 (47.76%)	332 (42.56%)	
T4	892 (18.50%)	248 (7.82%)	277 (31.80%)	367 (47.05%)	
AJCC *N*, 7th (2010-2015)					<0.001
N0	2607 (54.08%)	2607 (82.24%)	0 (0.00%)	0 (0.00%)	
N1	913 (18.94%)	494 (15.58%)	317 (36.39%)	102 (13.08%)	
N2	642 (13.32%)	67 (2.11%)	405 (46.50%)	170 (21.79%)	
N3	659 (13.67%)	2 (0.06%)	149 (17.11%)	508 (65.13%)	
Grade					<0.001
Well differentiated	515 (10.68%)	474 (14.95%)	27 (3.10%)	14 (1.79%)	
Moderately differentiated	1656 (34.35%)	1243 (39.21%)	258 (29.62%)	155 (19.87%)	
Poorly differentiated	2570 (53.31%)	1404 (44.29%)	577 (66.25%)	589 (75.51%)	
Undifferentiated; anaplastic	80 (1.66%)	49 (1.55%)	9 (1.03%)	22 (2.82%)	
Chemotherapy					<0.001
No/unknown	3362 (69.74%)	2434 (76.78%)	456 (52.35%)	472 (60.51%)	
Yes	1459 (30.26%)	736 (23.22%)	415 (47.65%)	308 (39.49%)	

*Abbreviations*: *LND* lymph node density, *OR* odds ratio, *CI* confidence interval

### Association between clinical variables and CSS and OS

**Fig. 2:**
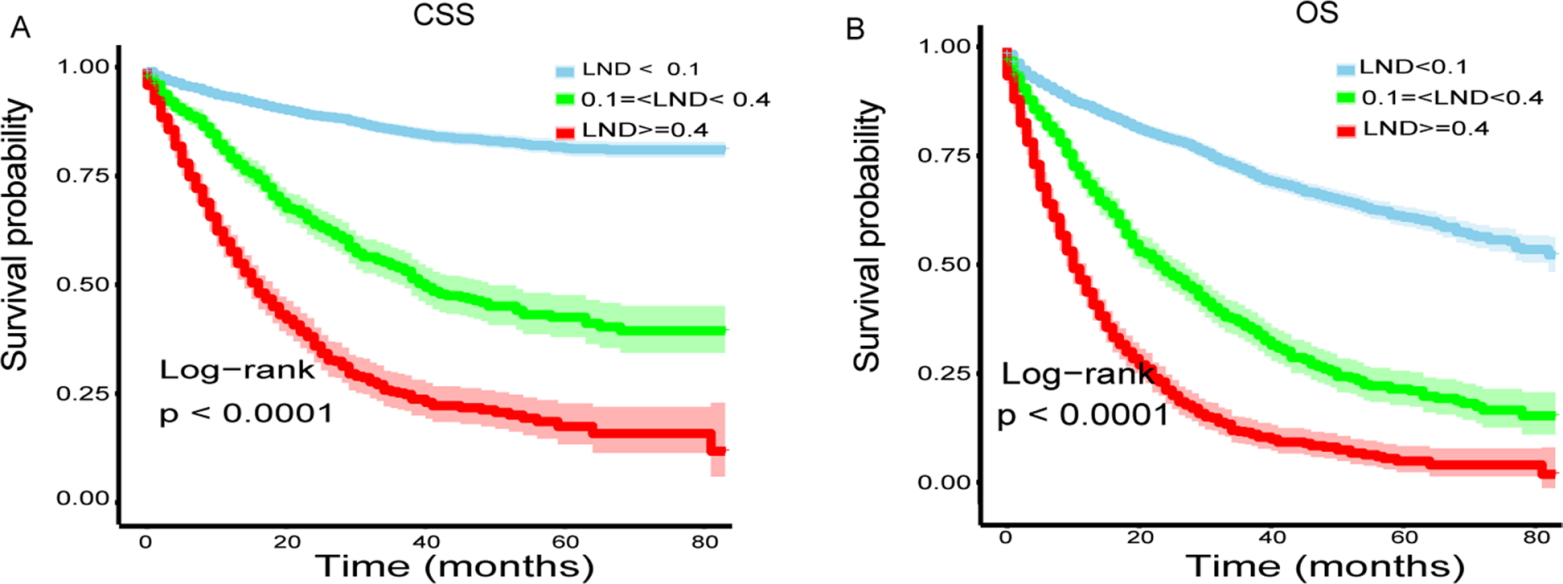
Kaplan–Meier survival curves of cancer-specific survival (**A**) and overall survival (**B**) for gastric cancer patients according to LND status

Table 2 shows the prognostic factors affecting CSS and OS in GC patients based on univariate analysis. Older age was significantly associated with reduced CSS (HR = 1.01, 95% CI 1.01–1.01, *P* < 0.001) and OS (HR = 1.03, 95% CI 1.02–1.03, *P* < 0.001). Null associations of sex were observed with CSS and OS. The mortality of GC patients was reduced with an increase in the year of diagnosis, although this result was just short of being statistically significant. The effect of race was also assessed, with Asian or Pacific islander populations showing a lower risk of mortality than white patients in CSS (HR = 0.79, 95% CI 0.68–0.92, *P* = 0.002) and OS (HR = 0.71, 95% CI 0.63–0.80, *P* < 0.001). Marital status at diagnosis was associated with GC survival and being widowed and single was associated with a dismal prognosis in CSS and OS analysis. Uninsured patients or those with unknown insurance status were at high risk of death in CSS (HR = 1.21, 95% CI 1.08–1.35, *P* = 0.001) and OS (HR = 1.13, 95% CI 1.03–1.23, *P* = 0.007). Compared to upper GC, primary tumors located at overlapping lesions significantly increased the risk of mortality both in CSS analysis (HR = 1.48, 95% CI 1.21–1.82, *P* = 0.001) and OS (HR = 1.36, 95% CI 1.16–1.60, *P* = 0.001). There was a significant positive correlation between advanced AJCC staging and survival both in CSS and OS (all *P* values < 0.001), which was in accordance with our clinical experience. In addition, patients with poorly or undifferentiated GC had an approximately doubled mortality risk compared to those with well-differentiated tumors. Patients receiving chemotherapy had a 1.33-fold increase in mortality risk in CSS analysis than those without chemotherapy or unknown chemotherapy status (HR = 1.33, 95% CI 1.19–1.50, *P* < 0.001), but the association was not significant in OS analysis. The primary variable in this study was LND and its survival predicting effects were fully investigated. Patients in LND2 or LND3 had 3.98 (95% CI 3.45–4.60, *P* < 0.001), 8.57 (95% CI 7.48–9.82, *P* < 0.001) folds increase in mortality risk in CSS, respectively, than those in LND1. Differences in mortality rate of OS were also shown for patients in the LND2 or LND3 which had 2.88 (95% CI 2.59–3.20, *P* < 0.001) and 5.54 (95% CI 4.99–6.15, *P* < 0.001) fold increase in mortality risk compared to those with the lower level. Kaplan–Meier survival curves for those patients according to LND status are presented in Fig. 2. Significant differences were observed among LND subgroups in predicting CSS (Fig. 2A, *P* < 0.001) and OS (Fig. 2B, *P* < 0.001).

**Table 2:** Association between clinical variables and gastric cancer-specific survival or overall survival

Variables	CSS, HR (95%CI) *P* value	OS, HR (95%CI) *P* value
Age at diagnosis (years)	1.01 (1.01, 1.01) < 0.001	1.03 (1.02, 1.03) < 0.001
Sex		
Male	Ref.	Ref.
Female	0.96 (0.86, 1.08) 0.536	0.92 (0.84, 1.00) 0.061
Year of diagnosis		
2010	Ref.	Ref.
2011	1.13 (0.95, 1.35) 0.151	0.99 (0.87, 1.13) 0.866
2012	0.90 (0.75, 1.08) 0.272	0.90 (0.78, 1.03) 0.121
2013	0.98 (0.81, 1.18) 0.841	0.95 (0.83, 1.10) 0.516
2014	0.91 (0.74, 1.11) 0.336	0.89 (0.77, 1.04) 0.141
2015	0.91 (0.72, 1.13) 0.384	0.79 (0.66, 0.94) 0.009
Race		
White	Ref.	Ref.
Asian or Pacific islander	0.79 (0.68, 0.92) 0.002	0.71 (0.63, 0.80) <0.001
Black	1.03 (0.87, 1.21) 0.740	1.02 (0.90, 1.16) 0.757
Other	0.76 (0.42, 1.38) 0.372	0.65 (0.40, 1.05) 0.079
Marital status at diagnosis		
Married	Ref.	Ref.
Widowed	1.40 (1.20, 1.63) < 0.001	1.52 (1.36, 1.71) < 0.001
Single	1.32 (1.12, 1.56) 0.001	1.26 (1.11, 1.43) 0.001
Other	0.99 (0.83, 1.18) 0.902	1.08 (0.95, 1.24) 0.242
Insurance		
Insured	Ref.	Ref.
Uninsured or unknown	1.21 (1.08, 1.35) 0.001	1.13 (1.03, 1.23) 0.007
Primary site		
Upper	Ref.	Ref.
Middle	0.96 (0.82, 1.11) 0.553	0.98 (0.87, 1.10) 0.699
Lower	1.03 (0.90, 1.19) 0.646	1.10 (0.98, 1.22) 0.093
Overlapping lesion	1.48 (1.21, 1.82) 0.001	1.36 (1.16, 1.60) 0.001
AJCC stage group, 7th (2010-2015)		
Stage I	Ref.	Ref.
Stage II	3.40 (2.79, 4.13) < 0.001	2.12 (1.87, 2.40) < 0.001
Stage III	9.75 (8.18, 11.62) < 0.001	4.80 (4.30, 5.35) < 0.001
AJCC T, 7th (2010-2015)		
T1	Ref.	Ref.
T2	1.99 (1.56, 2.55) < 0.001	1.44 (1.23, 1.69) < 0.001
T3	4.76 (3.96, 5.73) < 0.001	2.81 (2.50, 3.16) < 0.001
T4	10.10 (8.36, 12.20) < 0.001	5.16 (4.55, 5.85) < 0.001
AJCC N, 7th (2010-2015)		
N0	Ref.	Ref.
N1	3.35 (2.85, 3.94) < 0.001	2.27 (2.02, 2.55) < 0.001
N2	5.02 (4.24, 5.93) < 0.001	3.13 (2.77, 3.55) < 0.001
N3	9.14 (7.84, 10.67) < 0.00	5.32 (4.75, 5.96) < 0.001
Grade		
Well differentiated	Ref.	Ref.
Moderately differentiated	1.91 (1.42, 2.56) < 0.001	1.57 (1.30, 1.89) < 0.001
Poorly differentiated	3.96 (2.99, 5.23) < 0.001	2.46 (2.06, 2.95) < 0.001
Undifferentiated; anaplastic	3.82 (2.35, 6.20) < 0.001	2.64 (1.86, 3.74) < 0.001
Chemotherapy		
No/unknown	Ref.	Ref.
Yes	1.33 (1.19, 1.50) < 0.001	0.99 (0.90, 1.08) 0.776
LND		
LND1(< 0.1)	Ref.	Ref.
LND2(>= 0.1, < 0.4)	3.98 (3.45, 4.60) < 0.001	2.88 (2.59, 3.20) < 0.001
LND3(> = 0.4)	8.57 (7.48, 9.82) < 0.001	5.54 (4.99, 6.15) < 0.001

*Abbreviations*: *LND* lymph node density, *CSS* cancer-specific survival, *OS* overall survival, *HR* hazard ratio, *CI* confidence interval

### Independent prognostic effect of LND and stratified effect analysis in current lymph node staging systems

**Fig. 3:**
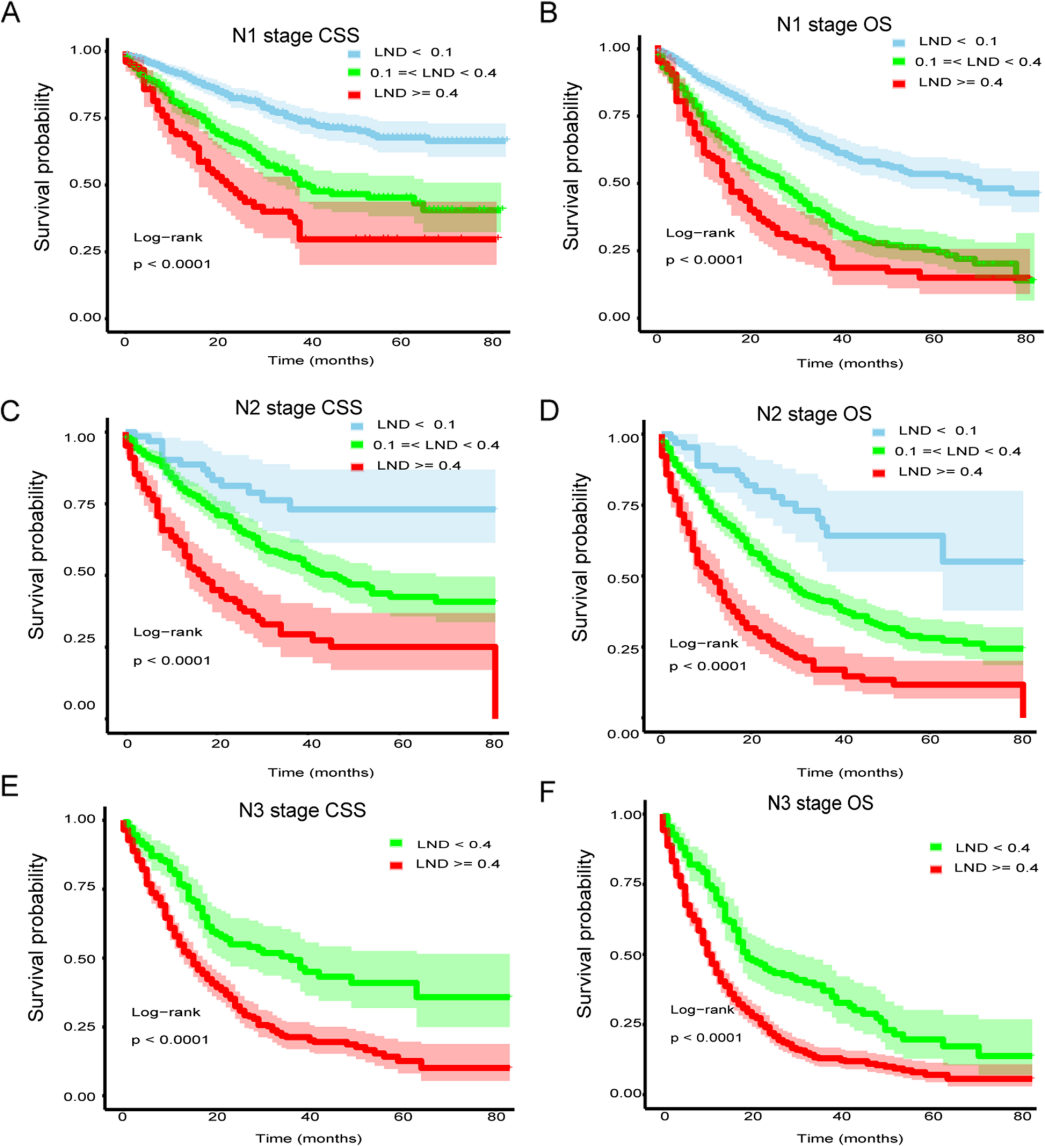
Stratified effect analysis of LND in positive N stages. Kaplan–Meier survival curves of cancer-specific survival in N1 (**A**), N2 (**C**), and N3 (**E**) subgroups. Kaplan–Meier survival curves of overall survival in N1 (**B**), N2 (**D**), and N3 (**F**) subgroups

To further elucidate the prognostic impact of LND on GC patients who underwent gastrectomy, Cox multivariate analysis was performed (Table 3). We constructed three models to analyze the independent effects of LND on survival including a crude (non-adjusted) model, minimally adjusted (adjusted I) model and fully adjusted (adjusted II) model. As previously shown in the results of multivariate analysis, CSS and OS of GC patients decreased with an increase in LND in different models. In the fully adjusted model, compared to the lower LND group, patients with a middle and higher level of LND had 2.43 (HR = 2.43, 95% CI 2.09–2.84, *P* < 0.001) and 4.69 (HR = 4.69, 95% CI 4.02–5.48, *P* < 0.001) fold increase in mortality in CSS, respectively. Similar results were found in the evaluation of OS which showed that patients with a middle and higher level of LND had 2.04 (HR = 2.04, 95% CI 1.81–2.29, *P* < 0.001) and 3.61 (HR = 3.61, 95% CI 3.20–4.07, *P* < 0.001) fold increase in mortality, respectively. Taken together, these data suggest that LND had independent prognostic effects in patients with GC. To further elucidate whether LND had independent prognostic effects in the current nodal category strategies, subgroup analysis was performed. Each positive N category (N1-3) was stratified into different LND subgroups. In the N1 and N2 stages, patients with GC were divided into three groups according to the cutoff points of LND. As shown in Table 3, CSS and OS of GC patients significantly decreased with the increase in LND grade in different models (all *P* values < 0.05). For patients in the N3 stage, there were only two patients in LND1 group (Table 1) and thus for patients in the N3 category the cutoff point was 0.4. Results repeatedly showed that a high level of LND was associated with worse survival in CSS and OS in different models (all *P* values < 0.05). In addition, survival curves were plotted using the Kaplan-Meier method to determine the independent prognostic effect of LND in subgroups of N categories. As presented in Fig. 3, patients in each positive N category were found to contain subgroups divided by LND, with significantly heterogeneous CSS and OS (all *P* values < 0.001).

**Table 3:** Multiple regression analysis to assess the independent effect in CSS and OS and stratified effect analysis in different N stages

LND	Non-adjusted		Adjust I		Adjust II	
	CSS, HR (95%CI) *P* value	OS, HR (95%CI) *P* value	CSS, HR (95%CI) *P* value	OS, HR (95%CI) *P* value	CSS, HR (95%CI) *P* value	OS, HR (95%CI) *P* value
Total (*n* = 4821)						
LND1(< 0.1)	Ref.	Ref.	Ref.	Ref.	Ref.	Ref.
LND2(> = 0.1, < 0.4)	3.98 (3.45, 4.60) < 0.001	2.88 (2.59, 3.20) < 0.001	3.97 (3.44, 4.58) < 0.001	2.84 (2.56, 3.16) < 0.001	2.43 (2.09, 2.84) <0.001	2.04 (1.81, 2.29) < 0.001
LND3(> = 0.4)	8.57 (7.48, 9.82) < 0.001	5.54 (4.99, 6.15) < 0.001	8.62 (7.52, 9.87) < 0.001	5.58 (5.03, 6.19) < 0.001	4.69 (4.02, 5.48) < 0.001	3.61 (3.20, 4.07) < 0.001
Subgroup analysis						
N1 (*n* = 913)						
LND1(< 0.1)	Ref.	Ref.	Ref.	Ref.	Ref.	Ref.
LND2(> = 0.1, <0.4)	2.23 (1.72, 2.88) < 0.001	2.21 (1.81, 2.69) <0.001	2.17 (1.67, 2.81) < 0.001	2.04 (1.67, 2.50) < 0.001	1.97 (1.51, 2.57) < 0.001	1.86 (1.52, 2.29) < 0.001
LND3(> = 0.4)	3.59 (2.58, 4.98) < 0.001	3.18 (2.44, 4.14) < 0.001	3.39 (2.43, 4.72) < 0.001	2.88 (2.21, 3.76) < 0.001	3.12 (2.21, 4.39) < 0.001	2.65 (2.01, 3.49) < 0.001
N2 (*n* = 642)						
LND1(< 0.1)	Ref.	Ref.	Ref.	Ref.	Ref.	Ref.
LND2(>= 0.1, < 0.4)	2.17 (1.25, 3.75) 0.005	2.46 (1.54, 3.92) 0.002	2.15 (1.24, 3.73) 0.006	2.44 (1.53, 3.90) 0.002	2.07 (1.19, 3.60) 0.010	2.34 (1.46, 3.76) 0.001
LND3(> = 0.4)	4.77 (2.72, 8.38) < 0.001	5.07 (3.13, 8.21) < 0.001	4.72 (2.68, 8.30) < 0.001	4.88 (3.01, 7.92) < 0.001	4.71 (2.63, 8.46) < 0.001	4.87 (2.96, 8.01) < 0.001
N3 (*n* = 659)						
LND < 0.4	Ref.	Ref.	Ref.	Ref.	Ref.	Ref.
LND > = 0.4	2.51 (1.87, 3.38) < 0.001	1.86 (1.50, 2.31) < 0.001	2.07 (1.60, 2.70) < 0.001	1.92 (1.54, 2.39) < 0.001	1.94 (1.49, 2.53) < 0.001	1.80 (1.44, 2.25) < 0.001

Non-adjusted model did not adjust covariant

Adjusted I model minimally adjusted for sex, age, and race

Adjusted II model fully adjusted for sex, age, race, marital status at diagnosis, insurance, AJCC Stage Group 7th (2010-2015), AJCC T 7th (2010-2015), and grade

*Abbreviations*: *LND* lymph node density, *CSS* cancer-specific survival, *OS* overall survival, *HR* hazard ratio, *CI* confidence Interval

## Discussion

Accurate staging is the cornerstone of optimal cancer care and therapy. The current staging system of N categories, the AJCC TNM system, for GC patients is based on the number of lymph nodes with metastases and the number of examined lymph nodes is not taken into account. The prognostic role of LND, calculated by the ratio of the number of positive nodes to the number of examined nodes, in GC staging was analyzed in the present study. Optimal cutoff values of LND were calculated as 0.1 and 0.4, and LND was divided into three levels: LND1 (< 0.1), LND2 (> = 0.1, < 0.4), and LND3 (> = 0.4). Significant differences in survival were observed among LND subgroups and in predicting CSS and OS by univariate analysis. We constructed three models to analyze the independent effects of LND on survival and results showed that LND had independent prognostic effects in patients with GC. To evaluate the performance of LND in the staging system, each N category was stratified by different levels of LND. Each N category was found to contain subgroups of GC patients with significantly heterogeneous CSS and OS.

Currently, the AJCC TNM system is the most commonly used staging system for GC. From the 7th edition AJCC staging manual, the N category was recommended to include resection of at least 16 regional lymph nodes [17, 21]. A considerable amount of literature has been published on the topic of LND or LNR from 2010, when the 7th edition AJCC staging manual firstly came into efforts [22, 23]. Many studies have demonstrated that regional positive nodes did not or insufficiently reflect the survival of patients with GC [14, 24, 25]. For patients who were detected with the same number of regional positive nodes, those with more regional nodes examined were more likely to have better survival because those patients tended to be with a higher level of radical gastrectomy and have lower risk with the residual tumor. Validation analysis from Taiwan University Hospital Cancer Registry also found the advantages of a staging system using a lymph node ratio using a patient cohort from Eastern medical centers [26]. One study from China showed that the lymph node ratio staging system is a reliable classification for GC patients after neoadjuvant radiotherapy [27].

In the present study, we demonstrated that LND was an independent predictor of patients’ survival and to have stratified effects in different N subgroups. Although these findings have already been reported in previous studies [18, 22, 25, 26, 28], and some of these also used data from the SEER database [18, 25], our study has distinct characteristics that make it differ from previous studies. The time of enrolment in the present study was from 2010 to 2015, during which period the 7th AJCC staging strategies were applied and the follow-up and treatment information was complete. Most previous studies enrolled GC patients across a long-time span, and patients before 2010 were also included for analysis. Another consideration is that, before the application of the 7th AJCC staging system, the number of examined lymph nodes may have been lower. A recent study by Yang et al. included GC patients from the SEER database between 1988 and 2015 and 61.31% of patients had less than 15 examined lymph nodes [18, 29]. LND or lymph node ratio is determined by the number of positive regional nodes and the number of examined regional nodes. With the development of surgical techniques, an increasing number of regional nodes can be resected and examined. Data before 2010 when the lower number of examined regional nodes was common may inevitably affect the lymph ratio and result in unstable evidence. In the present study, the median regional nodes examined were 17.96, which was higher than the 16 in the 7th edition AJCC staging system, thus providing a relatively higher level of evidence. Another distinct characteristic of our study is that we only enrolled patients with gastric adenocarcinoma according to histologic subtype codes of the SEER database. The significance of lymph node metastasis is different in different histologic types [30, 31]. Gastric adenocarcinoma comprises nearly 90% of the total number of gastric malignancies and thus focusing on adenocarcinoma helps to better understand the characteristics of this histologic subtype and improve diagnosis and treatment [32].

Although the present study is a relatively large population-based study, it inevitably had several limitations. First, the SEER database is a public open-access cancer registry data and does not include certain information regarding neoadjuvant chemotherapy and surgical complications which may influence the survival of GC patients. Second, the retrospective nature of the SEER database does not allow to extrapolate the results. Third, only 4281 patients were analyzed from a total of 17,988 gastric adenocarcinomas, due to incomplete data. Therefore, the conclusion of this paper is limited to gastric adenocarcinoma. Fourth, in this study, we used data from SEER database only, and lacked independent datasets from our center or other centers. Fifth, because the SEER database lacks information such as the extent of lymph node dissection (D0-2) and the curability (R0-2) of participants, these two factors were not fully considered in this study. Finally, the 8th edition of the AJCC TNM system has been recommended in clinical practice since 2017, but data from the SEER database from after 2017 cannot be used because of the limitation of lack of follow-up information.

In conclusion, by analyzing the SEER database from 2010, when the 7th edition of the AJCC TNM system came into efforts, the present study demonstrates that LND is an indicator of CSS an OS in GC. We calculated two optimal cutoff values of LND as 0.1 and 0.4. Significant differences in survival were observed among LND subgroups in predicting CSS and OS. Patients in each positive N category were found to contain subgroups divided by LND with significantly heterogeneous CSS and OS. Development of a new LND staging strategy may help improve patient care and to better informed and precise treatment decisions. However, due to the retrospective nature of data from the SEER database, further prospective cohort studies are needed.

## Conclusion

Through analysis of a population-based SEER database, we found that LND is an independent prognostic factor for GC without distal metastasis. In the current lymph node staging system, LND has potential value in further accurately classifying the GC patients without distal metastasis.

## Supplementary Information

**Additional file 1: Supplemental Figure 1.** Determining the optimal cutoff value of LND for predicting cancer-specific survival using x tile software. (A) Bar graph representing population distribution; (B) Kaplan–Meier survival curves divided by LND cutoff; (C) relative risk analysis among subgroups divided by cutoff value of LND.

**Additional file 2: Supplemental Figure 2.** Determining the optimal cutoff value of LND for predicting overall survival using x tile software. (A) Bar graph representing population distribution; (B) Kaplan–Meier survival curves divided by LND cutoff; (C) relative risk analysis among subgroups divided by cutoff value of LND.

## Data Availability

The data that support the findings of this study are available from the corresponding author upon reasonable request.

## Abbreviations

SEER: Surveillance Epidemiology and End Results
GC: Gastric cancer
LND: Lymph node density
CSS: Cancer-specific survival
OS: Overall survival
AJCC: the American Joint Committee on Cancer
TNM: Tumor-node-metastasis
