# Supplementary figures and images for: Prognostic value of lymph node density on cancer staging system for gastric cancer without distal metastasis: a population-based analysis of SEER database
Source: World J Surg Oncol. 2022 Sep 29;20:325. doi: 10.1186/s12957-022-02795-9 (PMC9520926; doi:10.1186/s12957-022-02795-9)

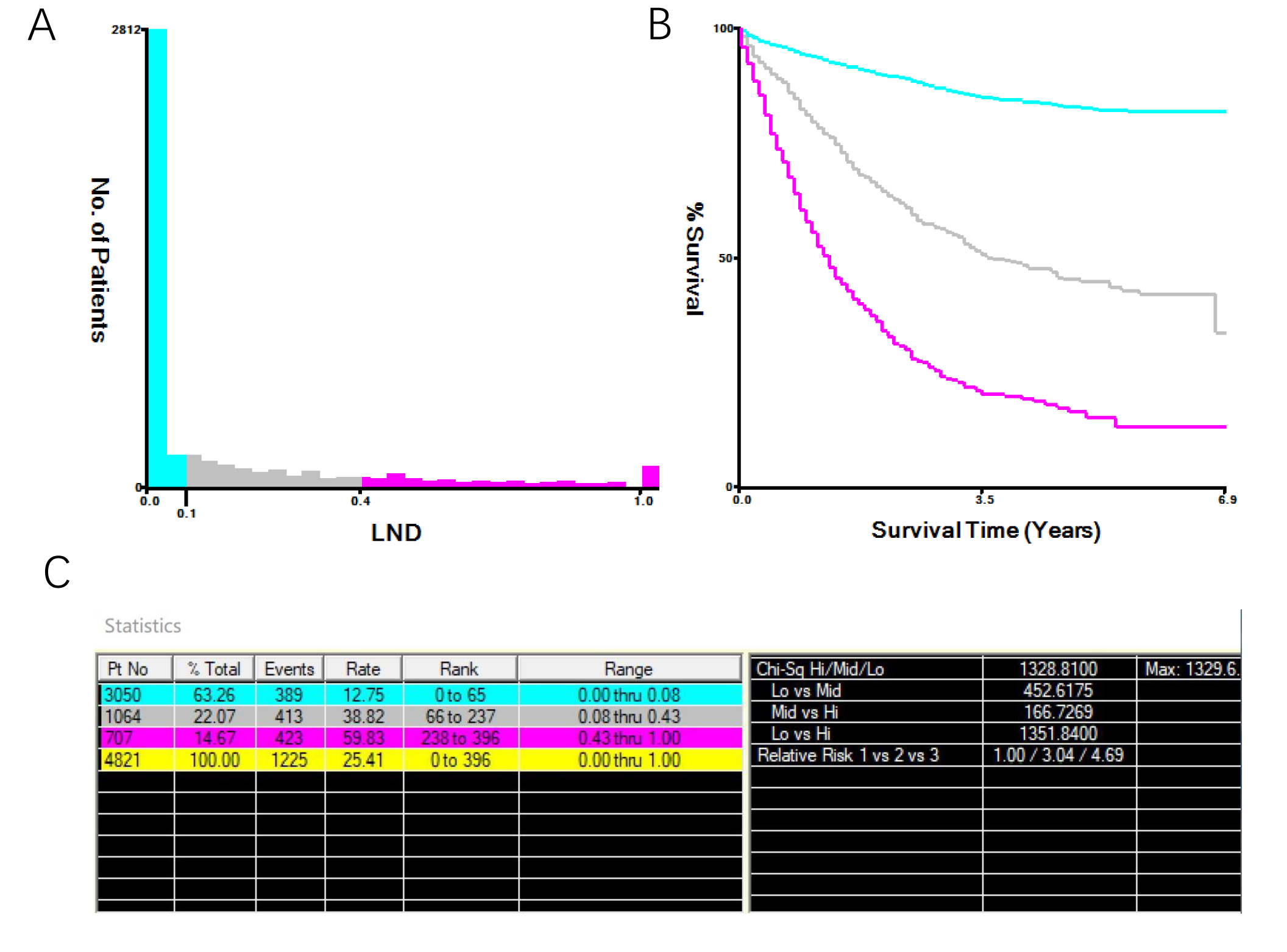

Supplement: Supplementary file 1 — Additional file 1: Supplemental Figure 1. Determining the optimal cutoff value of LND for predicting cancer-specific survival using x tile software. (A) Bar graph representing population distribution; (B) Kaplan–Meier survival curves divided by LND cutoff; (C) relative risk analysis among subgroups divided by cutoff value of LND. [file 12957_2022_2795_MOESM1_ESM.tif]

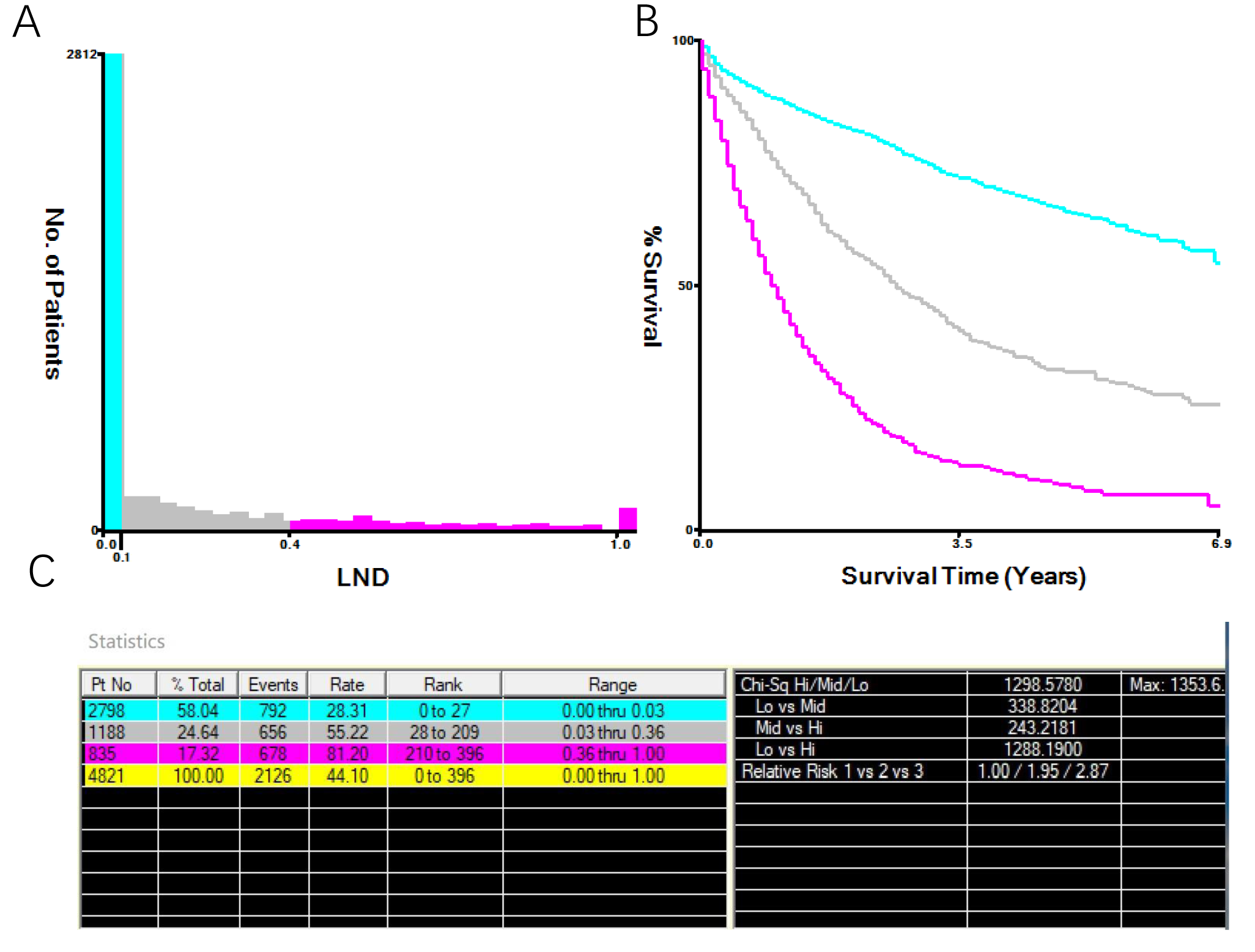

Supplement: Supplementary file 2 — Additional file 2: Supplemental Figure 2. Determining the optimal cutoff value of LND for predicting overall survival using x tile software. (A) Bar graph representing population distribution; (B) Kaplan–Meier survival curves divided by LND cutoff; (C) relative risk analysis among subgroups divided by cutoff value of LND. [file 12957_2022_2795_MOESM2_ESM.tif]
